# Recruitment of general practitioners in China: a scoping review of strategies and challenges

**DOI:** 10.1186/s12875-022-01854-0

**Published:** 2022-09-26

**Authors:** Shiwei Chen, Xin Hui Sam, Aijia Soong, Lorainne Tudor Car, Siqing Lian, Helen E. Smith

**Affiliations:** 1 Family Medicine and Primary Care, Lee Kong Chian School of Medicine, Nanyang Technological University, Singapore, 308232 Singapore; 2 Peking University First Hospital, Beijing, China

**Keywords:** Primary care, China, General practitioners, Recruitment, Family doctors, Healthy China 2030

## Abstract

**Background:**

China is rapidly expanding its general practitioner (GP) workforce as part of recent healthcare reform, with an extra 400,000 GPs by 2030. This scoping review identifies the published strategies for GP recruitment that are being implemented and the challenges encountered.

**Methods:**

We searched six English and three Chinese databases from 2015 to April 2022, following Arksey and O’Malley’s framework and the PRISMA ScR reporting guidelines.

**Results:**

A total of 40 Chinese-language and 5 English-language records were included. We identified multiple policies, pathways and programmes focused on expanding GP recruitment. Twenty-two evaluations of these initiatives show varying degrees of effectiveness. Selecting general practice as a career is affected by many factors, including individual’s background, remuneration and benefits, career prospects, working environment, self-fulfilment, and current national developments and reorganisations of primary care. The challenge most frequently reported was the adequate provision of qualified GP in rural regions. The targeting of students from rural areas and provision of free education in return for an obligatory six-years’ working in their hometown upon graduation appears to be effective. Extracted records mostly studied views of trainees in a defined locality, and we identified a paucity of studies which explored the perspectives of organisations and institutions, similarly there were areas of China not contributing to the literature and there were no records taking a national perspective.

**Conclusions:**

Long-term monitoring is required to assess policy changes and to systematically evaluate the effectiveness of the interventions nationally. The monitoring of the challenges influencing GP recruitment can be used to inform the design of future initiatives.

Development of a minimum agreed standardised set of outcomes used to measure and report evaluations will help assess the relative contributions and cost effectiveness of different approaches being used to boost GP numbers. We provide suggestions for improving the benefits and rewards for GPs and how to promote recruitment to the more rural or less attractive areas.

**Supplementary Information:**

The online version contains supplementary material available at 10.1186/s12875-022-01854-0.

## Background

There has been a rapid expansion of the general practitioner (GP) workforce in China in recent years. The number of GPs per 10,000 residents has nearly doubled from 1.38 in 2015 to 2.61 in 2019 [1, 2]. This rapid expansion is a result of the nationwide healthcare reform, with a focus on developing community health organizations and primary care to create a solid foundation for the hierarchical diagnosis and treatment system (fenji zhenliao zhidu) (HDTS) [3–5]. HDTS aims to divert the overcrowding and heavy demand at tertiary hospitals, particularly by patients seeking treatment for common conditions or chronic disease, to community healthcare institutions in order to tackle the issue of “being difficult and expensive to see doctors (kanbing nan, kanbing gui)”. It encourages patients to visit primary care for their first diagnosis (jiceng shouzhen) and establishes a two-way referral procedure (shuangxiang zhuanzhen) between primary, secondary and tertiary care, enabling patients who are recovering from treatment in tertiary hospitals or who have chronic conditions to be referred back to primary care institutions. This new emphasis on primary care in the medical system requires a large expansion of the GP workforce. The General Office of State Council announced the goal to increase the number of GPs to five per 10,000 residents by 2030, this requires an extra 400,000 GPs to be registered within the next decade [6]. The goal of increasing to 5 GPs per 10,000 population is moderate compared to the current ratios of other developed countries: South Korea 6.3, United Kingdom 7.5, Canada 13.1, and France 14.2 [7].

**Fig. 1:**
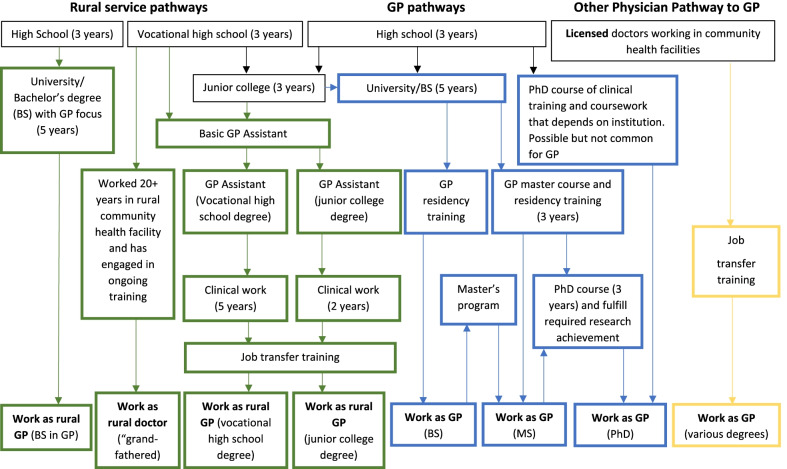
Three main pathways to work as GPs in China. (This is an adapted version of the original figure) Source: Lian S, Chen Q, Yao M, Chi C, Fetters MD. Training Pathways to Working as a General Practitioner in China. Fam Med. 2019;51(3):262–270. https://doi.org/10.22454/FamMed.2019.329090. Copyright Society of Teachers of Family Medicine. Used with permission

Previous research has evaluated interventions to promote recruitment in primary care and has identified challenges encountered, but these studies come mostly from Europe, Australia and USA [8–12], countries where primary care systems and training are already well established. In China the context is different, GP recruitment is being promoted in parallel with major reorientation of the health care system. The numerous new pathways to becoming a GP being developed in the country can be grouped into three main categories: rural service pathways, GP pathways and transfer of other physicians to the GP pathway [13]. Each pathway differs in its entry criteria, duration of training and the ultimate job eligibility (See Fig. 1). For example, the rural service pathways target students from high schools in rural regions and provide them with five years free education to become GPs, with an obligatory six-years’ service in their hometown upon graduation.

### Objectives

For an international audience to access, understand and learn from the strategies, challenges and experiences of GP recruitment in China, there is a need to collate the relevant evidence, as most records are written in Chinese only. Our objective was to systematically explore, identify and review the available literature on strategies, challenges and outcomes of the initiatives being developed in China to promote the recruitment of GPs.

## Methods

### Study selection

This scoping review was undertaken using the Arksey and O’Malley’s framework [14] and reported according to PRISMA extension for scoping reviews (PRISMA ScR) [15]. We chose to conduct a scoping review not a systematic review because the studies are heterogenous in nature. Our aim was to identify available evidence in the field, how research is conducted on this topic, and to identify and analyse knowledge gaps [16]. We focused on explicit strategies and their effectiveness, as well as the factors and challenges affecting recruitment. Studies published in Chinese or in English and regardless of study design were included. We focused only on studies related to GP recruitment and excluded studies that addressed doctors’ job satisfaction, job stress, career change, job confidence, attitudes towards job and job incentives, career development and generic professional training but without any discussion on how these factors influence GP recruitment. Similarly, we also excluded records focussing on recruitment strategies related to or brought about by policy reform or curriculum development but where there was no evaluation of their effectiveness to attract GPs. Studies of healthcare professionals that were multidisciplinary (e.g., allied health professionals, nursing, medicine, etc.) were included only if the data for GPs had been analysed separately. A protocol for this review was registered on The Open Science Framework (OSF) [17].

### Data sources, collection, analysis

A comprehensive search of the literature was conducted in the following databases: PubMed, Medline, Embase, Cochrane Library, PsycINFO, CINAHL, China National Knowledge Infrastructure (CNKI), VIP Information Network (CQVIP) and Wanfang. We searched the databases in May 2022 for relevant studies. The key search terms in English included general practitioner, recruitment, personnel management and China. Some adaptations were made for the search terms in Chinese. The full search strategy for English and Chinese records are listed in Additional files 1 & 2 respectively.

The search results from each bibliographic database were imported into EndNote X8.0.2 [18] to form a single combined library. After duplicates were removed three reviewers, proficient in both English and Chinese languages, independently screened the collated titles and abstracts and excluded records not related to the topic or about an irrelevant study population. Disagreements between reviewers were resolved through consensus-based discussion. Relevant citations were retrieved for full-text review. Three reviewers independently extracted data using a data extraction form and cross checked for missing information.

Data for each study was extracted as follows:

- Study reference—author, year of publication, study location or setting, study design (types and measurement tools), study aims
- Demographics of study population (number of participants, gender, age, education level, income, occupation, place of origin of participants (where available)
- Policy or programme evaluated
- Key findings (factors affecting recruitment, challenges)
- Recommendation and suggestions

Discrepancies in the extracted data were resolved through discussion between at least two reviewers. No formal quality assessment was performed on the included studies as the purpose of the review was to map available evidence rather than critically appraise.

### Data synthesis and analysis

We performed a qualitative content analysis [19] of key findings and categorized the data according to the main factors identified. Two reviewers identified and agreed on key factors, and these were then refined in discussion with the co-authors. Factors influencing and deterring recruitment were refined throughout the review process and reported according to frequency. Content analysis was supported by the use of NVivo 12 Plus [20].

### Patient and public involvement

No patient involved.

## Results

**Fig. 2:**
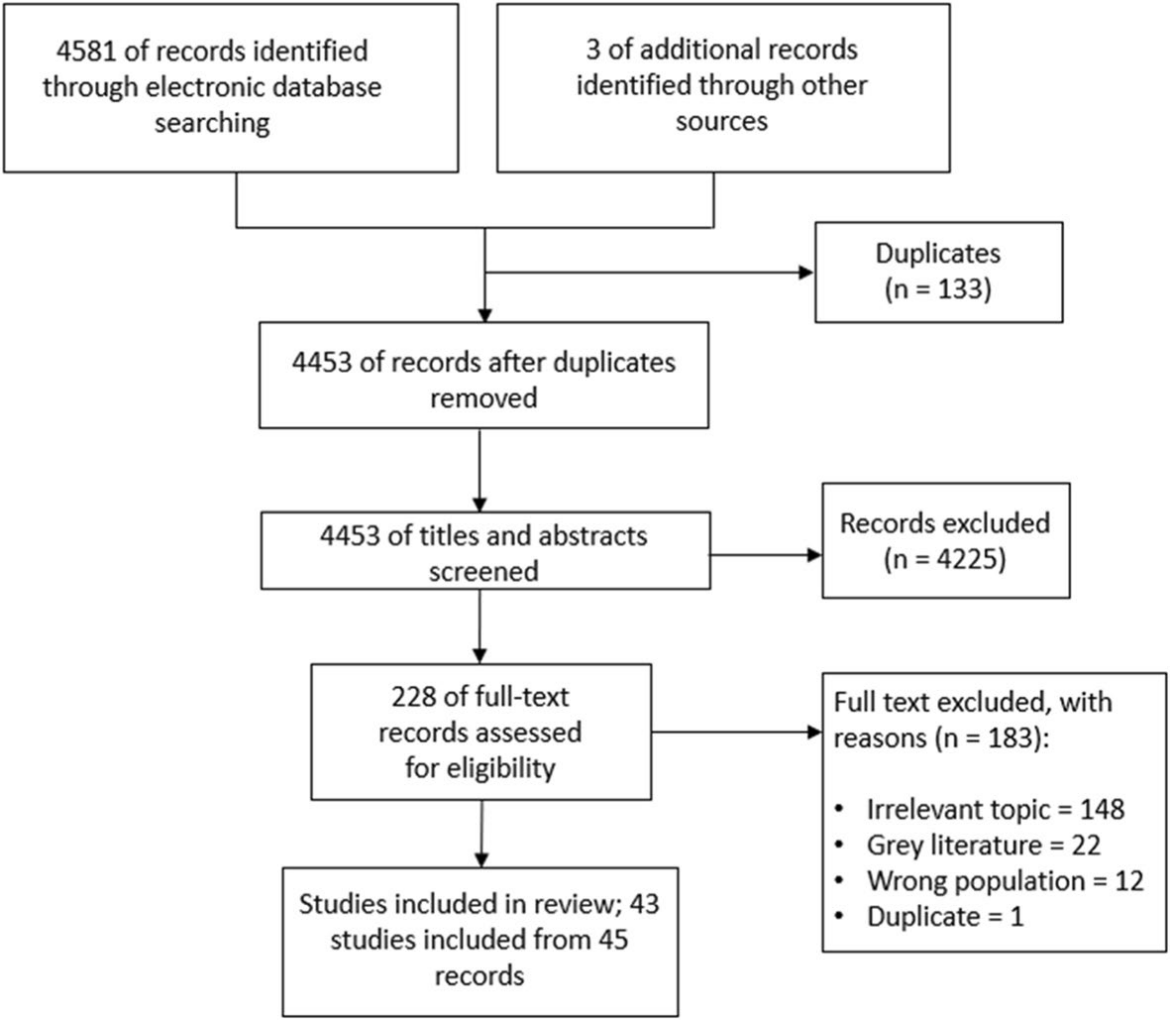
PRISMA flow chart of included studies. PRISMA, Preferred Reporting Items for Systematic Reviews and Meta-Analyses; GP recruitment strategies and challenges in China

Our search strategy identified a total of 4584 records (Fig. 2). After the removal of 133 duplicates, we screened 4453 records and identified 228 full-text records that addressed recruitment of GPs in China. From these 228 records, 182 were excluded as they failed to meet the inclusion criteria, leaving 45 works published between 2015 and April 2022. These 45 records (36 peer-reviewed journal articles, one paper from a conference proceeding, eight Master theses) referring to 43 studies. Forty records were written in Chinese (Study numbers (SN) 1–40 in Additional file 3) and 5 in English (SN 41–45).

Additional file 3 provides an overview of the 45 extracted records. Of the 43 relevant studies, 27 were quantitative studies (25 studies were surveys and two used institutional records), twelve were mixed-method studies (i.e., using both qualitative and quantitative data) and four were qualitative studies using individual interviews. A total of 18,893 participants were included in the 43 studies, sample size ranged from 12 to 2593 participants. In the 36 studies reporting gender, slightly more respondents were female (55.2%). Most participants were aged between 20 to 30. One study was conducted online and involved the whole of China and 36 studies reported on specific localities, including 15 provinces (Anhui, Guangdong, Guangxi, Guizhou, Hebei, Henan, Inner Mongolia, Jiangsu, Jiangxi, Liaoning, Shaanxi, Shandong, Sichuan, Yunnan, Zhejiang) and three direct-administered municipalities (Chongqing, Shanghai, Tianjin). Only four studies reported GPs’ income; one study in Chongqing published in 2015 reported that 91.6% had monthly income less than 4000 Yuan (approximately USD567), one study published in 2019 reported that 71.8% of GPs in Guangxi had a yearly income 50,000 Yuan (approximately USD7600) or less [21, 22]. Another more recent study in Hangzhou published in 2021 reported significantly higher monthly income with nearly half (46.8%) earning more than 8000 Yuan, which included 14.7% earning more than 15,000 Yuan [23].

Eleven studies included medical undergraduates, postgraduate students and/or graduates majoring in GP and a further 16 studies on medical undergraduates with a non-GP major. Of the studies on participants majoring in general practice, eleven studies focussed on trainees participating in five different GP training programs. One study investigated the impact of COVID-19 outbreak on intention to become GPs among trainees and the majority still decided to become GPs [24]. Extracted records usually focused on one of the three main pathways (Fig. 1); 29 records discussed the general pathways to becoming a GP [24–52], 13 records focused on the rural service pathways [21, 25, 27, 28, 35, 37, 50, 53–58] and 5 records reported findings on the career transfer pathway to GP [23, 25, 37, 41, 59].

### Overview of GP recruitment policy and programmes evaluated

22 records [21, 22, 33, 34, 36–41, 47, 52–62] referred to policies and/or programs developed to increase the recruitment rate to general practice. The policies were implemented at different administrative and institutional levels and organised by various health organizations and universities. Where available, we summarise the main strategies mentioned, and the evaluations reported in Table 1.

**Table 1:** Available GP recruitment strategies and corresponding evaluation studies

Year of Implementation	Policy/Intervention	Policy/Intervention content	Evaluations reported in extracted records
2014	*Guidance on establishing a standardized training system for GP residents* [34, 36–39, 41, 62]	A 3-year residency training program after graduation, which includes 3 months of general practice related theory, followed by 26-month rotation in clinical departments and a 7-month internship in primary care	Seven papers evaluated training programmes in Henan, Zhejiang, Shanghai and Yunnan. A training centre was established in 2015 at First Affiliated Hospital of Zhengzhou University in Henan [34]. Before the training, 187 respondents (83.5%) had no knowledge of general practice; after the training, 166 (83%) had a good understanding and 159 (95.8%) were willing to engage in the field. Among 51 graduates completing training, 26 found employment but only 8 (30.8%) chose to work as GPs. Another study on trainees in an unspecified training centre in Zhengzhou found that 97 (66.4%) were willing to become GPs [39]. Similarly, a study at Zhejiang University found that 55 (87.3%) trainees worked in general practice after training [41]. In contrast a study in Henan found that among 332 graduates from 56 medical organizations across the province, only 111 (36%) worked in general practice [38]. In another study, based in 11 training centres in Shanghai, [36] 165 (79%) trainees expressed willingness to become GPs after training [37]. In the Yunnan study, 43.3% trainees expressed willingness to work in general practice after the training
2010	Job-Transfer-to-GP Training [22, 59, 62]	One of the approaches to boosting the number of qualified GPs is the Job-Transfer-to-GP scheme. Licensed doctors and assistant GPs working in community health facilities and secondary or above hospitals who intend to become GPs are eligible to receive GP training at appointed training centres and are then assessed by provincial departments of health. Those that pass are eligible to register as GPs or assistant GPs. This scheme reduces the training time from three years to two years or less, to help with meeting the urgent need for GPs	Three papers reported outcomes of job-transfer training and showed that the programme was not effective in recruiting GPs. In Chongqing, 36 people signed up for the training but only six registered as GPs when the program ended. The remainder (83.3%) did not become GPs and reported reasons including that “it was not required by their employers”, “intend to register, but not taking action yet”, “did not meet registration requirement”, “having concerns about becoming a GP”, “no general practice in the current workplace” and “considering to resign from the current workplace” [22]. In the second study from Yunnan [59] among 282 job-transfer trainees, 198 were unwilling or unable to register as GPs. Over half failed to meet GP registration requirements (58.6%), 9.6% lacked confidence in GP career development, 8.1% were concerned about impact on future job transfer opportunities, 2.5% were unwilling to become GPs. Of the 84 progressing to work as GPs the primary reasons chosen by those who decided to register as GPs were willingness to become GPs (67.9%), job appointment requirements (15.5%), departmental administrative requirements (8.3%), preferential state policies (7.1%) and others (1.2%). Similarly, a study in an unidentified city in Henan found that between 2015–2019, more than 130 doctors completed either standardized training or job-transfer training, but only 45 registered as GPs
2014	Eight Incentive Measures [54]	The policy implemented by the regional government of Pudong New Area, Shanghai provides incentives to healthcare personnel including GPs working at 12 “farther rural” community health centres (CHCs), 11 rural CHCs, and 10 suburban CHCs to tackle the shortage of health personnel in rural areas. GPs working at farther rural, rural and suburban areas receive monthly incentive of RMB6000, 4000, 2000 (approximately USD 927, 618, 309) respectively. GPs moving to rural areas for at least 5 years receive a bonus of RMB150,000 (USD 23,170) or 200,000 (USD 30,894) according to their seniority	An institutional investigation on the quantity information of the GP’s inflow and outflow at CHCs in Pudong between 2012 and 2016 [54] found that before the incentive policy, the population of GPs in father rural and rural CHCs grew on average by 2.3 and 8.5% annually but after the introduction of incentives growth increased to 6.8 and 14.3%, confirming the benefit of the incentive policy on manpower growth
2011	Masters’ degree in GP: “5 + 2 + 1 joint medical education model for general practitioners” [63]	In 2011, Guangzhou Medical University launched a Master degree program, including five years undergraduate clinical education, two years training of clinical rotations in hospital and one year community healthcare training (reduced to six month in 2012 with the nationwide implementation of standardized training system for residents) [63]	The program recruited 45 students between 2012–2016, and 37 graduated. Among graduates, 31 were employed in secondary and tertiary hospitals (two in a department of general practice and 29 in emergency and internal medicine), one in health management and one in an urban community health service centre. Four were unemployed. Of eight students yet to graduate, only one was considering a career in GP. Semi-structured interviews with all 45 students found that the low success rate promoting General Practice was attributed to: the relatively low salary and poor working conditions of GPs (41, 91%); low social status; concerns about career prospects(45, 100%); not able to focus on medical work because of multiple roles including preventive medicine, public education etc. (42, 93%); the living conditions at the working locations are not desirable (43, 95%)
2010	*Rural-on-Demand-Oriented-bonded-GP Training Programme* [21, 53, 55–58, 60]	Students enrolled in this five-years programme are predominantly recruited from rural areas, and priority is given to those from areas designated most in need of health care. Students are exempt from tuition and accommodation fees and central government provides 6,000 Yuan (approximately USD913) per year for living expenses. On course completion graduates are obligated to serve in a rural primary medical and health institutions for a minimum of 6 years or pay a penalty	Six studies [21, 53, 56–58, 60] [55] from Guangxi, Zhejiang, Yunnan, Anhui and one unspecified locations report on the outcome of this programme. Three studies [21, 56, 57] reported that the majority of the students were willing to or did become GPs after graduation. One study reported that only one out of 380 graduates broke the contract [55]. A longitudinal study in Anhui reported 97.5% of graduates between 2015 and 2017 served their bond at the designated workplace. In another study, from an unspecified location, [53, 60] found that less than half of the students were willing to work at primary care. A study from Zhejiang [57] described 17 graduates out of 91 breaching the contract because of difficulties improving their professional skills in primary care (58.9%), unpromising career prospects (52.9%), a lack of sense of achievement (47.1%), unsatisfactory remuneration and poor working conditions (41.2%)
Unknown	Including general practice courses in the curriculum for undergraduate medical students [33, 40, 47, 61]	At some medical schools, the traditional curriculum has been expanded to include a module *“Introduction to General Practice”* or offer practicing opportunities in general practice to boost students’ understanding of general practice. For example, in Shangqiu Medical College second year students have 16 h of theoretical teaching and 8 h of practical education on this topic [33]	Two survey-based studies [33, 47] evaluating the impact of broadening the curriculum found that students undertaking the “Introduction to General Practice” course had different perceptions of general practice. In Shangqiu Medical College [33] 82% of students who took the course thought that GPs had career development potential compared to 76% amongst controls. More students in the intervention group (70.5%) were willing to work in primary care institutions after graduation, compared to controls (61.5%). Another survey study [47] from Nanjing University of Chinese Medicine showed that after including the “Introduction to General Practice” into the curriculum 30% of the students were willing to consider working at a primary health care institution. Of the others, the majority (67.3%) felt that they could not make a decision until later, and a minority was explicitly unwilling (2.4%). The study from Shanghai University of Traditional Chinese Medicine found that among students who took courses in general practice, only 14 (12%) considered participating in standardized training to become a GP as their first career choice after graduation, 83 (71%) will participate in the trainning only if they do not have other options, and 20 (17%) expressed unwillingness to become GPs [40]

### Influencing factors and prominent challenges of GP recruitment

Extracted studies reported numerous influencing factors in GP recruitment, which appear to be common between three main pathways, and we summarise them into six main categories: the Individual’s Background, Remuneration and Benefits of GPs, GP Career Prospects, Working Environment of the General Practitioner, Self-fulfilment, and the National Developments and Reconfigurations of Primary Care (Table 2). Most extracted records (*n* = 35, 77.8%) addressed issues related to Remuneration and Benefits. The theme Working Environment included the most number of influencing factors.

**Table 2:** Influencing factors of GP recruitment

Main themes	Sub-themes	Examples
Individual’s background	Personal characteristics and family background (14 records) [32, 34–36, 40, 42, 47, 49, 55, 56, 58–60, 64]	Participants’ perceptions of GP as an occupation, intentions to serve the patients in their hometowns, financial background, family members’ suggestions in career pursue and views of general practice
	Qualifications (8 records) [23, 38–40, 44, 59, 60, 65]	Years of service, academic degree level, academic performance, professional titles, understanding of medical technology/medical services, different academic years of students
	Personal interest (14 records) [24, 30, 36, 38, 40–44, 46, 51, 53, 58, 60]	Academic interest in medicine/general practice/primary care
Remuneration and benefits	Income and benefits (35 records) [23, 25–30, 33–50, 52, 56–59, 61–65]	Low wages
	Living condition (1 record) [59]	Living in the rural regions
Career Prospects	Job development (27 records) [23, 25, 27–31, 33, 34, 36–42, 44–48, 51, 57, 58, 61, 63, 64]	GPs job scope and future development in comparison with specialists, career options, intention to work in large hospitals, aspiration for academic positions, accumulation of practice experience
	Training (8 records) [27, 28, 34, 36, 37, 45, 50, 57]	Foreseeable training opportunities, satisfactions of training participation experiences, desire to develop knowledge and competencies, attainment of certifications
	Job stability & employability (11 records) [36, 39, 40, 43, 44, 46, 48, 51, 56, 60, 64]	The amount of job openings, ongoing contracts
Work Environment	Working environment & conditions (12 records) [23, 27, 28, 32, 39, 40, 42, 46, 47, 53, 57, 58]	Collegial relationships, organizational management
	Outdated facilities [27, 29, 32, 44, 53, 63, 64] (7 records)	Outdated or lacking equipment
	Working location (11 records) [28, 32, 33, 36, 39, 45, 48, 58, 60, 62, 64]	Geographical locations
	Heavy workload (8 records) [27, 29, 30, 36, 40, 50, 62, 64]	Lacking manpower
	Work stress (7 records) [27, 30, 36, 39, 43, 51, 64]	Long working hours
	Doctor-patient interaction (6 records) [28, 30, 40, 45, 62, 63]	Safety issues and concerns in the workplace
Self-fulfilment	Job satisfaction (10 records) [28, 34, 42, 43, 45, 57, 58, 61, 62, 65]	Low sense of fulfilment at work
	Personal values (6 records) [23, 39, 42, 48, 62, 63]	Passionate about devoting to primary care development
	Social recognition (14 records) [23, 27–29, 33, 34, 36, 39, 40, 45, 48, 50, 63, 64]	Social status and recognition of GP as an occupation
National development and reconfigurations of primary care	Policy support (15 records) [23, 29, 34, 36, 37, 39, 40, 42–45, 56, 58, 60, 62]	Understanding of national policies
	Administration and management (5 records) [30, 38, 41, 45, 61]	Underdeveloped organizational structures

The important findings within each of these six themes are described in more detail below.

#### Individual’s background

Qualifications of trainees in Job-Transfer-to-GP Training program affect their unwillingness to register as general practitioners, and the proportion of trainees unwilling to register as GPs increased with their years of previous work experience and with higher academic qualifications [59]. Personal background and interests are factors that participants who are more knowledgeable about general practice and medical technology used in general practice, and with a more service-oriented personality have more optimistic anticipations of incomes and present higher job recognition, which is associated with higher willingness to become GPs [65]. Unfortunately negative personal experiences discourage students to become GPs, one study observed that as students progressed through their undergraduate training it was those who had more family medicine internship opportunities who became less enamoured with general practice as a career option, largely attributed to the “negative emotions” they observed in their GP clinical tutors [60].

#### Remuneration and benefits

Seven studies found that more than half of the respondents think the wage of GP is low, and it is one of the main reasons that they are unwilling to become GPs [27, 30, 36, 38, 48, 50, 63]. One study [59] interviewed 45 GP-major Master students and 43 expressed concern that primary care health institutions are usually in rural regions with poor socioeconomic development, which in turn adversely affects their living condition, as well as limiting the opportunities for their family.

#### Career prospects

The experiences of the training provided influenced willingness to become GPs; one study found that standardize training experience offered was suboptimal and discouraged trainees to become GPs [45]. Several studies highlighted that the job responsibilities of a GP were unclear to students, and this, together with numerous uncertainties about the career pathway, deterred students from becoming GPs [35, 46, 50, 52, 60]. One study [61] reported that 61.7% GP-major undergraduate students considered that the career prospects of general practice to be good, but less than a quarter (23.5%, 8/34) of them chose to be employed in general practice. The remainder preferring to change their career path to nutrition, health management, other medical-related majors, or non-medical-related majors, whilst some remained undecided.

#### Work environment

Medical students were discouraged from becoming GPs as the profession involved working at the grassroots, with outdated facilities and equipment [48, 52]. Institutions in rural areas faced additional challenges when trying to recruit GPs [32, 48]. Eleven studies [28, 32, 33, 36, 39, 45, 48, 58, 60, 62, 64] mentioned the importance of geographical location – with preferences being expressed for working in the participants’ hometown, in large hospitals, or big cities; remote places, other than their hometowns were undesired. Six studies reported poor doctor-patient interactions [28, 30, 40, 45, 62, 63] leading to personal safety issues and concerns in the workplace. One study reported positively on the desirability of the stable, long-term patient relationships that GPs can enjoy [62].

#### Self-fulfilment

Passion and a desire to develop primary care positively affected the willingness to become GPs [23, 27–29, 33, 34, 36, 39, 40, 45, 48, 50, 62–64]. In contrast, the lack of social recognition and minimal respect from the patients and the community adversely affected any willingness to complete three years of standardized GP residency training and pass national exam in order to register as GPs.

#### National development and reconfigurations of primary care

One study on medical students found that the better the understanding of national supportive policies in primary care, the more expressed willingness to work in primary care [60]. Two studies found that supportive national policies toward general practice were amongst the most important factors in choosing a career [37, 62]. In contrast, the reasons offered for excluding GP as a career on graduation were insufficient information and understanding about the national primary care policies for developing and strengthening the health care system [36] this was accentuated when trainees had questions about the policies, but they did not have a channel to obtain answers [39]. Some medical students and standardize training program trainees were not confident in, or satisfied with, the implementation of supportive policies [44, 45].

An obstacle to GP recruitment was the under-developed organization of the primary care system, evidenced by the lack of general practice in some medial institutions, or that after standardized training, graduates were assigned by their institutions to other specialities [38]. This under-development fuelled students concerns about the poorly defined role of GPs within Chinese healthcare [30]. Additionally, trainees were aware that GPs working at the community healthcare centres had limited prescription choices in comparison with tertiary hospitals under the national essential medicine system and this deterred their willingness to progress to register as a GP [31]. They were concern that when they were in the community, they would be unable to retain their patients because they lacked access to appropriate medications, which in turn would reduce the patient’s trust in them: “Drugs are limited in the community, when some patients visited, they asked for medicine they got previously from tertiary hospitals, but we don’t have those in the community [clinics], including antibiotics. I am qualified [to make the prescription], but I am not allowed to use [the drugs] on the patients” [31]. They also commented that “each household contracting [pairing] with one family doctor is a good policy, but no one taught me how to implement, the government didn’t provide a concrete plan” [31].

## Discussion

This is the first comprehensive review of GP recruitment strategies and challenges in China. Forty-five records were identified, with 40 published in Chinese. We found recruitment strategies that had been developed by regional healthcare or education institutions in response to state policies and guidelines. Six interventions identified focussed on increasing the total number of GPs and one of these was designed to also achieve a more even distribution of GPs between urban and rural regions. As yet some of the training programmes appear not to have achieved their desired impact on GP expansion. There were challenges implementing national policy at regional institutions, for examples some institutions were unable to provide sufficient financial support for their staff to participate in the GP training pathways [22, 50, 54]. One study described how participating in a training programme required the clinician to reduce the number of hours they practice clinically, which in turn leads to a decrease in pay and forfeiting of performance bonus, discouraging doctors’ enthusiasm to be part of a new training programme [25]. Only one article addressed the perspectives of institutional management; the question of why some healthcare institutions are unable to support these training programmes needed for successful GP recruitment and primary care development needs further investigation. This is a knowledge gap that needs to be closed if policy is to be implemented, overcoming unintended consequences and barriers.

We further analysed the factors and challenges that influence GP recruitment reported in the extracted studies, including individuals’ background, remuneration and benefits, career prospects, work environment, self-fulfilment as well as the national development and reconfiguration of primary care system. The improvement of these identified areas will help to attract more people to work as GPs and the ongoing expansion of the GP workforce in China. Over three quarters of the records referred to the theme Remuneration and Benefits, often highlighting apparent differences in pay scales across the country. Given variations in GP roles and different costs of living in different localities, national standardisation of pay may be impractical or inappropriate, but information on the rational for these differences may increase professionals understanding and acceptance of apparent disparities in pay. We also recommend developing other forms of compensations for GPs, such as longer holiday with allowances, sabbaticals in urban areas for rural GPs, and training grants, to increase the benefits of working in the community.

Improving working environments and social status of GPs is crucial to make general practice a more attractive occupation, and one approach would be to make general practice a formally recognised specialisation to increase the social status, prestige, and income of GPs, as well as creating greater trust among patients reassured by GPs longer and higher standard of training [66]. It is important that the training is also tailored to what are readily available in primary care. For example, the pharmacopeia is decided by the state in China. Primary care institutions and tertiary general hospitals differ in what medicines they are allowed to prescribe. Initiatives that lengthen GP training, so that it becomes comparable with the training of hospital specialists, may enhance the appeal of general practice but it is not a practical solution when the country urgently needs to increase the number of generalists. However, a longer term objective should be the development of general practice as a specialty, together with formalised continuing professional development, reaccreditation and the development of GPs with special fields of interest.

Many extracted records reported that the social status of GPs is low, and this is also observed in other countries [67]. Besides improving work conditions, remuneration and moving toward specialisation of GPs, increasing social recognition of the GP important roles is crucial. One approach is to promote a positive image of GPs in the media, increasing the public’s understanding of their role and making the profession more appealing [68].

The challenge in GP recruitment is not only increasing the total number of GPs, but also achieving the appropriate geographical distribution. Unfortunately regional disparity is significant in China: in 2018, central and western regions had only 1.45 and 1.93 GPs per 10,000 population, lower than the national average 2.2 [61] with recruitment of GP to rural areas being particularly problematic [11, 69–72]. Attracting GPs to work in rural areas has also challenged other large countries, and studies from Australia and Canada report a positive correlation between rural origin, rural placements during training with choosing to work in rural regions [72, 73]. The availability of electives in rural general practice for all medical students in China may stimulate early interest in rural medicine as a career possibility.

The Australian and Canadian studies referred to above also observed persisting barriers to rural workplace selection, including “remaining located far from family and friends over an extended period”, “few social and recreational activities to enjoy” and insufficient collegial support [69, 70]. China has promoted rural practice by encouraging students from rural areas to be recruited on GP training schemes, through provision of free medical education and stipends. After graduation, they are obligated to return to their hometown to work as GPs for six years, and encouragingly it seems that many of the graduates on these schemes do become GPs [21, 53, 55–58, 60]. As yet the retention rates and career trajectories after their bonded period remain largely unknown. Informed by effective approaches used in other countries, [72–74] and combining with concerns reported in extracted studies, we suggest there is a need to be attentive to infrastructural development in the rural regions to improve both clinical and recreational facilities to retain GPs in rural areas. Benefits beyond salary may help GPs continue to work in remote and rural areas, for example housing subsidies and educational opportunities for their children in a neighbouring urban region.

Another unknown is the longer term impact of having different pathways to becoming a GP in rural and urban areas, with the former requiring lower entry requirements to embark on training [13]. This could ultimately impact on the range and quality of the primary care provided and patient outcomes in the rural regions. Monitoring performance indicators and health outcomes will be needed to ensure there is geographical equity in health care provision. Initiatives to develop the ‘clinical courage’ needed for remote medical practice could be introduced during undergraduate training and sustained once doctors are in the field [75]. The formation of special interest groups and research networks among rural GPs could strengthen collegiate connectivity, academic stimulation and service development.

We found seven reviews [11, 12, 71, 72, 76–78] on GP recruitment challenges and strategies encompassing evidence from 21 countries (the USA, Canada, Chile, Brazil, Trinidad and Tobago, the UK, Germany, Norway, Greece, Australia, New Zealand, Japan, Malaysia, Iraq, Jordan, Lebanon, Pakistan, Saudi Arabia, Turkey, Ghana and Kenya). There were some concerns and factors regarding choosing general practice as a career outlined by these studies that were not addressed in our extracted records, such as personality, desire for a career that is stimulating, intellectually challenging and interesting [12] (e.g. continuity of care, benefits the patients, “lower preference for medical versus social problems” [78], the duration and quality of clinical exposure in GP during medical training [12, 77, 78] (e.g. their dedication, size of staff, existence of role models, composition) and “having family physician mentors”. Other potential strategies that were not mentioned in the extracted studies included improving physicians’ well-being, developing peer support initiatives, actively promoting GP as a career, and having specialised recruiters or case managers [11]. These may be worth further investigation in the Chinese context. Furthermore, in these countries mentioned above the concerns were focused on job flexibility (e.g. part time options or time off for travel and recreation, work in more than one specialty, labour mobility, earning flexibility, functional flexibility) and independence/autonomy [78]. In contrast in China, respondents showed more concerns about job stability and employability, and it is possibly related to the fact that the current development plan of primary care is relatively new and the career prospects are sometimes unclear [11, 12, 71, 72, 76–78].

### Strengths and limitations

Strengths of this review include the use of validated scoping review methodology, an extensive and systematic search across multiple databases and websites in both English and Chinese to maximise the number of Chinese records identified. Many of the reviewed studies were published in Chinese, and this is a unique article that can provide useful information to an international audience about current GP development in China. It is a timely review to address the urgent issue of GP recruitment for developing primary care that is promoted by the Chinese health authority. When stated, the response rate of surveys was often very good, ranging from 48 to 100% (20 studies had 100% response rate, 16 studies had 91-99%; 3 studies had 80–89% response rate). This high rate of response suggests that data is representative, and respondents were motivated to respond by an incentive or a strong personal relationship with the person administering the questionnaire. Records describing policies for increasing GP recruitment, but without evaluation or discussion of effectiveness were excluded. However, we took note of the policies and interventions they mentioned and compared with our extracted records to ensure the relevant policies had been included in our review. We continuously updated our data extraction sheet during the review process to ensure the final data extraction was comprehensive.

A limitation of this review is that we were unable to comment on the periodic policy review as our search of the literature identifies no relevant records, if they do exist, they appear not readily accessible in the public domain. The sharing of policy review findings in publications could generate ideas for future interventions and research to evaluate impact on GP recruitment. A limitation of this scoping review is the absence of any formal quality assessment of the included records but observed that the quality of some of the included articles was suboptimal. There were some papers that were relatively short and lacking detail. Therefore, we compared findings from these articles with more rigorous studies and confirmed their conformity before presenting their data.

### Implications for future research

China has the opportunity to evaluate and compare many different recruitment strategies (Fig. 1) to identify which are most cost effective, enabling resources to be allocated for optimal results. As yet, there is no evaluation of the impact on recruitment of career pathways which include higher degree education, for example where GPs with bachelor’s degree pursue a Master degree, or where undergraduates and Master graduates pursue a PhD. Furthermore, as China is becoming more and more interconnected globally, future research may look into emerging or potential international collaboration opportunities, such as recruitment of GPs from other countries [11]. The positive corelation between willingness to become GPs and “having volunteered to work in a developing country” is interesting and ways to incorporate overseas experiences of students and young doctors need to be explored [78]. The adoption and implementation of national policies are not uniformed across the country, the perspectives of management and the challenges they face when implementing national policies in their healthcare institutions may need further attention. The included studies mostly used quantitative surveys, but to achieve more in-depth insights into some of the recruitment challenges mentioned requires qualitative methods, the data could then inform the initiatives devised to reduce the perceived deterrents to careers in general practice. In addition, various strategies for recruitment of GPs should be assessed using robust evaluations. We did not identify studies focusing on challenges and strategies of recruiting GPs in every province (e.g., Xinjiang, Tibet, Gansu, Ningxia and Qinghai were not featured). There may of course be unpublished evaluations, but their absence in the public domain, reduces accessibility, scholarly communication, and opportunities to share regional experiences with a wider audience. The development of a website for facilitating the exchange of information on GP recruitment more informally than via publication could maximise the impact of all the evaluative efforts being undertaken. It may also act as the forum for defining a common dataset of core outcome measures to be used when evaluating GP recruitment and retention. Future research needs to move from descriptive to interventional, developing and evaluating novel interventions and strategies to tackle the emerging barriers to GP recruitment identified in this scoping review.

## Conclusion

Our review illustrates China’s determination to develop a large and trained general practice workforce through a variety of national and local strategies designed to improve the status of GPs, promote general practice as a profession, increase remuneration and welfare, and facilitating many different GP training pathways. Existing evaluations were mostly case studies, and the effectiveness of these strategies on GP recruitment on a national scale is unclear. Ongoing evaluation is needed to fully assess the impact of different policies, as many of them were introduced recently and evidence of their longer-term impact and sustainability of a larger GP workforce will only become apparent in the years to come. As China is undergoing rapid reforms and numerous initiatives are at an early stage, cross regional evaluation with methodological uniformity and common outcome measures will enable robust comparisons to be made. The identified factors summarized in this review can be used to inform the design of future research on GP recruitment and provide an analytical framework from a Chinese perspective.

## Supplementary Information

**Additional file 1. **

**Additional file 2. **

**Additional file 3. **

## Data Availability

Data are available on reasonable request. Please contact Shiwei Chen (schen021@e.ntu.edu.sg).

## References

[1] Department of Development Planning (2016). 2015 China health development statistical report.

[2] Department of Development Planning (2020). 2019 China health development statistical report.

[3] Wang H, Gusmano MK, Cao Q (2011). An evaluation of the policy on community health organizations in China: will the priority of new healthcare reform in China be a success?. Health Policy (New York).

[4] Li X, Lu J, Hu S, Cheng KK, De Maeseneer J, Meng Q (2017). The primary health-care system in China. Lancet.

[5] Healthy China 2030 (from vision to action). World Development. 2016. Available from: http://www.nhc.gov.cn/guihuaxxs/s3585u/201907/e9275fb95d5b4295be8308415d4cd1b2.shtml. [cited 23 Mar 2020].

[6] Zhan Q, Shang S, Li W, Chen L (2019). Bridging the GP gap : nurse practitioners in China. Lancet.

[7] OECD.Stat. Health care sources : physicians by categories. Available from: https://stats.oecd.org/Index.aspx?QueryId=30173. [cited 23 Mar 2020].

[8] Van Ham I, Verhoeven AH, Groenier KH, Groothoff JW, De Haan J (2006). Job satisfaction among general practitioners: a systematic literature review. Eur J Gen Pract.

[9] Groenewegen PP, Hutten JBF (1991). Workload and job satisfaction among general practitioners: a review of the literature. Soc Sci Med.

[10] Shadbolt N, Bunker J (2009). Choosing general practice: a review of career choice determinants. Aust Fam Physician.

[11] Verma P, Ford JA, Stuart A, Howe A, Everington S, Steel N. A systematic review of strategies to recruit and retain primary care doctors. BMC Health Serv Res. 2016;16(1). 10.1186/s12913-016-1370-1 10.1186/s12913-016-1370-1 PMC4828812 27067255

[12] Marchand C, Peckham S (2017). Addressing the crisis of GP recruitment and retention: a systematic review. Br J Gen Pract.

[13] Lian S, Chen Q, Yao M, Chi C, Fetters MD (2019). Training pathways to working as a general practitioner in China. Fam Med.

[14] Arksey H, O’Malley L (2005). Scoping studies: towards a methodological framework. Int J Soc Res Methodol Theory Pract.

[15] Tricco AC, Lillie E, Zarin W, O’Brien KK, Colquhoun H, Levac D (2018). PRISMA extension for scoping reviews (PRISMA-ScR): checklist and explanation. Ann Intern Med.

[16] Munn Z, Peters MDJ, Stern C, Tufanaru C, Mcarthur A, Aromataris E. Systematic review or scoping review ? Guidance for authors when choosing between a systematic or scoping review approach. BMC Med Res Methodol. 2018;1–7. 10.1186/s12874-018-0611-x 10.1186/s12874-018-0611-x PMC6245623 30453902

[17] Chen S, Soong AJ, Tudor Car L, Smith HE. General practitioner recruitment and retention in China: a scoping review protocol. Open Sci Framew. 2020.

[18] EndNote. EndNote X8.2 for Windows. Thomson Reuters; 2018.

[19] Sandelowski M (2000). Focus on research methods whatever happened to qualitative description ?. Res Nurs Health..

[20] QSR International Pty Ltd. NVivo qualitative data analysis software. 2018.

[21] Liao S, Yao Y, Liang Y, Li N, Chen J (2019). Current situation of targeted-area graduates of general practice in Guangxi province. Chinese Gen Pract.

[22] Wan X. Status of Position Transition Training For General Practice in Chongqing. Chongqing Medical University; 2015.

[23] Qin S, Ding Y (2021). Who is willing to participate in and provide family doctor contract service?: a cross-sectional study based on the medical staff’s perspective in China. Medicine (Baltimore).

[24] Yin Y, Chu X, Han X, Cao Y, Di H, Zhang Y (2021). General practitioner trainees’ career perspectives after COVID-19: a qualitative study in China. BMC Fam Pract.

[25] Lu Y. The study on general practitioners analysis and development in Chongqing. Chongqing Medical University; 2015.

[26] Yan W, Gao X, Lu Z (2018). Investigation of the perception towards general practice and career intention among clinical medicine undergraduates. Heal Vocat Educ.

[27] Zou J, Zhou M. Study on the Willingness and Influencing Factors of Medical Undergraduates to Apply for General Practitioners. Med Educ Res Pract. 2018;26(6):970–4. Available from: https://www-jstor-org.libproxy.boisestate.edu/stable/25176555?Search=yes&resultItemClick=true&searchText=%28Choosing&searchText=the&searchText=best&searchText=research&searchText=design&searchText=for&searchText=each&searchText=question.%29&searchText=AND

[28] Huang M, Qiu K, Bao R, Zhuang H, Bao S, Luo J (2016). General medical students career confidence index in China. Chinese J Gen Pract.

[29] Zhang S, Gao T, Tu X, Li S (2019). Analysis on the understanding and employment of general practitioners at the primary level under the new situation. Think Tank Era.

[30] Yuan S, Wang M, Peng M, Hou L, Lin Z, Wu G (2019). Investigation and analysis on general practice cognition and teaching guidance of medical students in colleges and universities. J Chem Inf Model.

[31] Li Y, Ji G, Yuan Q, Lu X, An J (2019). Barriers for general Practioner trainees becoming registered general Practioners: a survey. Chinese J Gen Pract.

[32] Xia H, Pan Z, Wang T, Wang J, Gu J, Yang H (2019). Survey on employment intention of medical students in China. Chinese J Gen Pract.

[33] Wang S (2020). Investigation on influence of general family medical education on employment direction of students in a medical college. Chinese J Sch Dr.

[34] Gao Y, Liu X, Xu H, Li Y, Guo Y, Zhao S (2020). Investigation and analysis of the first batch graduated general practice trainees from large general hospital in Henan province. Chinese Gen Pract.

[35] Zhang L, Bossert T, Mahal A, Hu G, Guo Q, Liu Y (2016). Attitudes towards primary care career in community health centers among medical students in China. BMC Fam Pract.

[36] Zhu J, Feng Y, Yuan T, Duan H, Shi R (2015). Investigation of theoretical training and employment intention of trainees in standardized training for residency of general practitioners in Shanghai. Chinese Gen Pract.

[37] Song J (2017). The research on the status and countermeasure in standardized training of general practitioners in Yunnan Province.

[38] Liu X X, LI B B, Miao Y Y, Wang S S, Xu H H, Wang L L (2020). Status of general practice residency training and career choice of trainees in Henan Province. Chinese Gen Pract.

[39] Zhao Q. A survey on the status of the perception of general practitioner occupation and employment Intention of “5+3” general practice resident trainees. The People’s Hospital of Zhengzhou University; 2020.

[40] Huang P, Li M, Yu L, Shi R (2021). Investigation on general practice sequence education model for traditional Chinese medicine undergraduate clinical students. J Tradit Chinese Med Manag.

[41] Chao G, Weng C, Zhu Y, Fang L. Status and influencing factors of general practice resident doctors after graduating from standardized training. China High Med Educ. 2020;12:18–19.

[42] Xie J, Liu Y, Na J, Zhao Y, Bian L (2020). Influencing factors on intention to become GPs of clinical undergraduate students at Hebei Medical University and analysis of strategies. J Hebei Med Univ.

[43] Chen Y, Wang Y, Hu J, Guo L, Sha F, Liu Y (2021). Clinical undergraduate students’ career intention to work at primary care and influencing factors. J Mod Med Heal.

[44] Ling L, Yao-hui Y, Xiao-fei X, Si-si X, Ya-ming L, Lin Y (2020). Investigation and study on the orientation and service willingness of TCM students. J Jiangxi Univ TCM.

[45] Yang H. Qualitative study on the factors influencing the practice intention of the trainees in standardized training of general practitioners in Shandong province. Shandong University; 2020.

[46] Shang L. Investigation of the cognition of general practice and intention of grass-roots work for the partial undergraduate medical college students in Yunnan. Kunming Medical University; 2016.

[47] Wang P, Gu Q. Investigation on the recognition and employment intention of the course “General Practice Introduction” in a Chinese medicine school. In: The inaugural meeting of the General Practice Branch of the Chinese Society of Chinese Medicine and the 2016 academic annual meeting. Shanghai, China; 2016.

[48] Luo L, Xu T (2016). Investigation on Medical students’ perception and career intention of general practice in a medical univesity. Heal Prot Promot.

[49] Liu Z, Ma J (2017). Analysis of the influencing factors of general practitioner career intention of undergraduates in a medical college in Tianjin. China Heal Stand Manag.

[50] Liu L. Status quo and countermeasures of general practiotioners’ practices in Chongqing. Chongqing Medical University; 2017.

[51] Wei H, Li F (2018). An analysis of the career intention to become GP and the influencing factors among medical undergraduates. Mod Med Heal Res.

[52] Liu L, Wu S, He M, Li X, Wang J, Cao Z (2018). Analysis of factors influencing the career choice of medical undergraduates to serve in primary care. Public Manag.

[53] Xue F, Qian R (2019). Analysis on the present situation and influencing factors of grassroots employment intention in rural order-oriented medical students. J Bengbu Med Coll.

[54] Jing L, Liu K, Zhou X, Wang L, Huang Y, Shu Z (2019). Health-personnel recruitment and retention target policy for health care providers in the rural communities: a retrospective investigation at Pudong new area of Shanghai in China. Int J Health Plann Manage.

[55] Shen Y, Huang X, Li H, Chen E, Kong Y, Yu J (2021). Early outcomes of a rural-oriented physician education programme against rural physician shortages in Guangxi province: a prospective cohort study. BMJ Open.

[56] Ren L, Ma B, Gao L. The present situation and effect of the training of rural-oriented medical students in a University in Anhui Province. J jiujiang Univ (natural Sci. 2018;(2):106–8.

[57] Zhang Y, Ma M, Ni H, Xu L, Su Y, Zhou E (2020). Investigation on the career development of general practitioners at the grassroot level: taking the first batch of rural-on-demand-oriented-bonded-GP in Zhejiang province as an example. China High Med Educ.

[58] Yuan D, Huang Q, Wu H, Sun M, Shu Q, Chen Y (2019). Analysis on occupational identity and influencing factors of rural demand-oriented medical students. Med Educ Res Pract.

[59] Li W, Shu Q, Huang Q, Jiang Y, Yuan D, Zhao H (2018). The intention and influencing factors of registration of practitioners in the training before transferring the post of general practitioners in Yunnan Province. Chinese J Gen Pract.

[60] Xue F, Zhao J (2018). Analysis on the present situation and influencing factors of primary care employment intention in rural-oriented medical students. J Mudanjiang Med Univ.

[61] Ge W, Chen W, Li C, Xue M, Han Y, Ma X (2019). Investigation and analysis on occupational cognition of undergraduates majoring in general practice. Chinese Med Ethics.

[62] Zhao D. Training and development issues of primary care general practitioners: City X in Henan as an example. Minzu University of China; 2020.

[63] Wei D, Feng X, Zhang C, Gu Y, Cao X (2018). Causes and countermeasures of disconnection between training and practice of general practitioners in China. Chinese Gen Pract.

[64] Rui Z, Liu F (2016). Investigation of the cognition of speciality and career intention of undergraduates in general practice medicine. J Bengbu Med Coll.

[65] Song Y (2017). Career cognition and career intention among general practitioners in China : the mediating effect of income expectation and career identity. Chinese J Heal Policy.

[66] Barber S, Brettell R, Perera-Salazar R, Greenhalgh T, Harrington R (2018). UK medical students’ attitudes towards their future careers and general practice: a cross-sectional survey and qualitative analysis of an Oxford cohort. BMC Med Educ.

[67] Norredam M, Album D (2007). Prestige and its significance for medical specialties and diseases. Scand J Public Health.

[68] Barry E, Greenhalgh T (2019). General practice in UK newspapers: an empirical analysis of over 400 articles. Br J Gen Pract.

[69] Economist. Shod, but still shoddy – China needs many more primary-care doctors – but memories of barefoot ones put some people off seeing them. Economist. 2017.

[70] Primary health care in China. Lancet Reg Heal - West Pacific. 2020;3:100019. 10.1016/j.lanwpc.2020.100019 PMC8315346 34327380

[71] Brooks RG, Walsh M, Mardon RE, Lewis M, Clawson A (2002). The roles of nature and nurture in the recruitment and retention of primary care physicians in rural areas : a review of the literature.

[72] Viscomi M, Larkins S, Gupta TS (2013). Recruitment and retention of general practitioners in rural Canada and Australia: a review of the literature. Soc Rural Physicians Canada.

[73] Dunbabin JS, Levitt L. Rural origin and rural medical exposure: their impact on the rural and remote medical workforce in Australia. Rural Remote Heal. 2003;3(1):1–17. 15877502

[74] Jones MP, Humphreys JS, Nicholson T (2012). Is personality the missing link in understanding recruitment and retention of rural general practitioners?. Aust J Rural Health.

[75] Konkin J, Grave L, Cockburn E, Couper I, Stewart RA, Campbell D (2020). Exploration of rural physicians’ lived experience of practising outside their usual scope of practice to provide access to essential medical care (clinical courage): an international phenomenological study. BMJ Open.

[76] Bunker J, Shadbolt N. Choosing general practice as a career - the influences of education and training. Aust Fam Physician. 2009;38(5):341–4. 19458806

[77] Senf JH, Campos-outcalt D, Kutob R. Factors related to the choice of family medicine : a reassessment and literature review. 2003;16(6):502–12. 10.3122/jabfm.16.6.502 14963077

[78] Puertas EB, Arósquipa C, Gutiérrez D. Factors that influence a career choice in primary care among medical students from high-, middle-, and low-income countries: a systematic review. Rev Panam Salud Publica. 2013;34(5):351–8. 24553763

