# Supplementary material for: Recruitment of general practitioners in China: a scoping review of strategies and challenges
Source: BMC Prim Care. 2022 Sep 26;23:249. doi: 10.1186/s12875-022-01854-0 (PMC9511450; doi:10.1186/s12875-022-01854-0)
Supplement: Supplementary file 1 — Additional file 1. [file 12875_2022_1854_MOESM1_ESM.docx]

| 1     general practitioners/ or physicians, family/ or physicians, primary care/ (4619)  2     (GPs or general physician$ or general practitioner$ or general practice physician$ or family practitioner$ or family physician$ or family doctor$ or primary care practitioner$ or primary care doctor$ or primary care physician$).ab,ti,tw. (25284)  3     exp Personnel Management/ (17873)  4     exp Organizational Policy/ (927)  5     exp "Attitude of Health Personnel"/ (22056)  6     exp job satisfaction/ (3565)  7     (recruitment$ or recruitment strateg$ or personal recruitment$ or personnel recruitment$ or retention strateg$ or personnel retention$ or personnel turnover$ or professional development$).ab,ti,tw. (38944)  8     exp China/ (59802)  9     1 or 2 (26558)  10     3 or 4 or 5 or 6 or 7 (77844)  11     8 and 9 and 10 (33) |
| --- |

**Appendix 1: Search strategy for English Articles**
