# Supplementary material for: Recruitment of general practitioners in China: a scoping review of strategies and challenges
Source: BMC Prim Care. 2022 Sep 26;23:249. doi: 10.1186/s12875-022-01854-0 (PMC9511450; doi:10.1186/s12875-022-01854-0)
Supplement: Supplementary file 2 — Additional file 2. [file 12875_2022_1854_MOESM2_ESM.docx]

**Appendix 2: Search Terms for Chinese Articles**

| **Chinese** | **English translation** |
| --- | --- |
| 全科医生, 全科医师 | General practitioners |
| 家庭医生,家庭医师 | Family doctors |
| 初级保健医生,初级保健医师 | Primary care doctors |
| 社区医生,社区医师 | Community doctors |
| 就业 | Employment |
| 倦怠 | Burnout |
| 需求 | Need |
| 职业信心 | Employment confidence |
| 医学生 | Medical students |
| 职业满意度 | Job satisfaction |
| 职业认同 | Job recognition |
| 离职 | Turnover |
| 培养 | Nurturing |
| 激励 | Incentives |
| 奖励 | Rewards |
| 转岗 | Job-transfer |
| 吸引力 | Attractiveness |
| 招聘 | Recruitment |
| 职业选择 | Job selection |
| 工作积极性 | Work Motivation |
| 收入 | Income |
| 待遇 | Remuneration |
| 职业发展, 职业生涯 | Career development |
