# Supplementary material for: Recruitment of general practitioners in China: a scoping review of strategies and challenges
Source: BMC Prim Care. 2022 Sep 26;23:249. doi: 10.1186/s12875-022-01854-0 (PMC9511450; doi:10.1186/s12875-022-01854-0)
Supplement: Supplementary file 3 — Additional file 3. [file 12875_2022_1854_MOESM3_ESM.docx]

**Appendix 3: Overview of Extracted Articles**

In compiling this table, we used the English abstract and the title of an article if one is provided. Where there were inaccuracies in translation, we have also included a revised version.

* Some Chinese articles include an English abstract and title in the journal publication, and we adopted the original English text where appropriate. However, when the English translation was inaccurate, we have amended and indicated the change in square brackets [xxxx].

| **SN** | **Year** | **Study Title** | **Study Design**  **(Details are always included if provided)** | | **Study aims** | **Study population** | | ***N***  ***(Valid responses)*** | |
| --- | --- | --- | --- | --- | --- | --- | --- | --- | --- |
| **Chinese articles** | | | | | | | | |  |
| 1 | 2018 | ^*^The present situation and effect of the training of Rural－Oriented Medical Students in a University in Anhui Province[44] | | Survey and interview | To study students’ enrolment motivation, understanding of the free training, employment intention and employment status, and use the summarised experiences to identify areas for improvement to increase the impact of the policy. | | Rural-on-Demand-Oriented-Bonded-GP medical graduates from three cohorts 2012(n=44), 2013(27), 2014(35) at Anhui College of Traditional Chinese Medicine | | 106 |
| 2 | 2017 | ^*^Career cognition and career intention among general practitioners in China: The mediating effect of income expectation and career identity[52] | | Survey of random sample | To explore how an understanding of GP influences career intention and career pathways. | | Pool of job-seeking doctors applying for jobs in the recruitment portal in a city of Shandong Province | | 304 |
| 3 | 2017 | ^*^Analysis of the influencing factors of general practitioner career intention of undergraduates in a medical college in Tianjin[37] | | Multi-stage sampling method, questionnaire surveyed on (1) demographics and (2) career intention (21 indicators) | ^*^To investigate the general practitioners’ career intention and the [its] influencing factors in a medical university in Tianjin. | | Undergraduates in a medical school in Tianjin enrolled on a 5-year medical degree course. Students were sampled from 3 classes within years 1, 2, 3 and 4. | | 298 |
| 4 | 2018 | An analysis of the career intention to become GP and the influencing factors among medical undergraduates[39] | | Multi-stage sampling method, questionnaire surveyed (1) Willingness to become GPs with 21 indicators and (2) student demographics | To explore the career intention of medical undergraduates to become GPs and the influencing factors. | | Undergraduates enrolled on a 5-year medical course in a medical school in Xi’an | | 500 |
| 5 | 2018 | Analysis of factors influencing the career choice of medical undergraduates to serve in primary care[40] | | Cluster sampling for survey, questionnaire surveyed (1) demographics and (2) medical students’ understandings and willingness to work at primary care institutions | To understand the intention to work in primary care and the influencing factors among medical undergraduates. To propose strategies to attract and retain medical students in primary healthcare facilities. | | Medical undergraduates in the North China University of Science and Technology | | 319 |
| 6 | 2018 | Investigation of the perception towards general practice and career intention among clinical medicine undergraduates[41] | | Cluster sampling for survey, questionnaire surveyed on (1) demographics, (2) career intentions, (3) understandings and attitudes toward general practice, (4) career expectations of general practice and (5) free text answer to the question: what are the current deficiencies of general practice in China? | To understand the perceptions of general practice and the career intentions of medical undergraduate majoring in clinical medicine | | Clinical medicine undergraduate from Cohorts 2010-2013 | | 2593 |
| 7 | 2015 | ^*^Status of position transition training for general practice in Chongqing[21] | | Literature research on job transfer to GP in both Chinese and English publications, cluster sampling for survey, interview | ^*^To know the status of position transition training for general practitioner in Chongqing and put forward corresponding views and suggestion for problems, in order to formulate the scientific development planning of position transition training for general practitioner and promote the development of this career. | | Survey was distributed to programme trainees, trainees’ colleagues who did not participate in the training, and patients of the participants.  Interviews with programme trainer, programme administrator, participating programme trainees | | 146 trainees responded to the survey;  31 trainees were interviewed |
| 8 | 2017 | ^*^The research on the status of countermeasure in Standardized GP Training Programme in Yunnan Province[33] | | ^*^Literature review, routine data collection and questionnaire survey, personal interview and group interview. | ^*^To investigate the current status of Standardized GP Training Programme in Yunnan Province. Analysing the process and influencing factors through the perspectives of organisers and trainees. Then putting forward suggestions to improve the level of GP’s standardized training in Yunnan, and hopefully, providing scientific reference for relevant policy. | | Trainees of Year 2014 and 2015, together with trainers and administrators of the programme. | | 219 trainees |
| 9 | 2015 | ^*^The study on general practitioners analysis and development in Chongqing[22] | | Literature review and secondary data analysis based on official statistics, survey and interview including demographic characteristics, training opportunities in general practices and career satisfaction | To understand the quantity, quality and component of GPs in Chongqing, in order to clarify problems in GP recruitment and propose strategies to tackle the issues. | | Survey was distributed to practitioners working at primary care; Interviews with health administrators, authorities at the community health service centres and doctors in township hospital | | 192 practitioners |
| 10 | 2017 | ^*^Status quo and countermeasures of general practitioners’ practices in Chongqing[38] | | ^*^Questionnaire and interview outline were designed after literature review, stratified cluster sampling for questionnaire, deep interview | ^*^To explore and analyse the main problems and influential factors in general-practitioner-building project in Chongqing by learning about the conditions of GP registration, professional cognition, practicing enthusiasm, status quo of GP training, demands and feedback.  To provide references and realistic basis for relevant departments’ further exploration of a proper operation mechanism of GP’s group construction and enhancement of the GP’s building in local hospitals by making practical suggestions. | | Qualitative interview: one GP who participated in GP training, one administrator and the person-in-charge from the relevant district and county health and family planning commission from all 58 primary health care institutions.  Quantitative survey: clinicians from all 58 primary care institutions who are on duty on the day of survey administration. | | 137 former general practice trainees, including 52 registered GPs |
| 11 | 2016 | ^*^Investigation of the cognition of general practice and intention of grass-roots work for the partial undergraduate medical college students in Yunnan[34] | | ^*^Stratified cluster sampling method for self-compiled questionnaire | ^*^To know the recognition of general practice, the intention of grass-root work and the reasons why they want or do not want to be a general practitioner in the partial undergraduate medical college students and discuss the relevant factors affecting the intention of being a practitioner since general practice education course was offered in Yunnan medical colleges. To provide basis for medical colleges to improve general education further and cultivate more general practice talents. To provide reference for the governments to develop general practice, attract and retain general practice talents better. | | 1925 medical undergraduate which include year 1 (647), year 2 (626) and year 3 (638) students who are studying in these specialties, clinical medicine, preventive medicine, rehabilitation medicine, medical imaging, anaesthesiology and traditional Chinese medicine. | | 1911 |
| 12 | 2018 | ^*^The intention and influencing factors of registration of practitioners in the training before transferring the post of general practitioners in Yunnan Province[47] | | Stratified cluster sampling for survey designed by the authors and interview | ^*^To understand the intention and influencing factors of practitioner registration in the transfer training for general practitioners in Yunnan province. To make suggestions for improving the registration intention of the general practitioner and to provide reference for the relevant departments to promote the construction of the grassroots doctors’ team. | | 300 primary medical and health workers who are participating in the Job-Transfer-to-GP Training from 6 counties of Yunnan Province | | 282 |
| 13 | 2019 | ^*^Current situation of targeted-area graduates of general practice in Guangxi Province[20] | | Survey designed by authors to investigate basic information of respondents, registration and transfer status, employment, implementing staffing, job satisfaction, willingness to receive further education, willingness to serve at primary care, salary expectancy; in-depth interviews | ^*^To provide suggestions on the reform and development of community-level general practice through analysing and discussing major constraints based on the status quo investigation of targeted-area graduates from general practice major in Guangxi Province. | | A total of 199 targeted-area students who were enrolled from 2010 to 2012 and graduated between 2015 and 2017. | | 199 |
| 14 | 2015 | ^*^Investigation of theoretical training and employment intention of trainees in standardized training for residency of general practitioners in Shanghai[23] | | ^*^Self-administered survey on basic information, purpose attending the training, understanding about general practice, effect of the training, time arrangement of the training, employment intention and corresponding reasons | ^*^To investigate the theoretical training and employment intention of trainees in standardized training for residency of general practitioners in Shanghai and to explore effective ways to improve the quality of the training. | | Three cohorts (2010, 2011, 2012) of trainees of GP standardized training program provided by 11 training bases (Ruijin Hospital, Xinhua Hospital, Shanghai First People's Hospital, Shanghai Third People's Hospital, Shanghai Sixth People's Hospital, Tongji Hospital Affiliated to Tongji University, Fengxian District Central Hospital, Zhabei District Central Hospital, Jiading District Central Hospital, Xuhui District Central Hospital and Gongli Hospital) | | 209 |
| 15 | 2019 | Analysis on the understanding and employment of general practitioners at the primary level under the new situation[26] | | Cluster sampling for two surveys designed by the authors after conducting literature review | To study undergraduates’ understanding of general practitioners and analyse whether undergraduates’ employment intentions have changed after learning systematically general practitioner basic knowledge and related policies. To explore the relationship between having the correct understanding of general practice and choosing general practice as a career, as well as other factors that affect their employment intentions. The study aims to provide new ideas for promoting general practice to medical students and the standardising training of general practitioners. | | Clinical medicine undergraduates studying clinical medicine (five-year program) (N=200) (76.1%), clinical medicine (“5+3” integration program) (N=62) (23.6%), and clinical medicine (undergraduate and master integrated program) 1 undergraduate (0.4%) | | First survey: 263; second survey: 220 |
| 16 | 2020 | Investigation on the career development of general practitioners at the grassroot level: taking the first batch of Rural-on-Demand-Oriented-bonded-GP in Zhejiang Province as an example[45] | | Questionnaire designed by the authors was distributed online and in person, interview | Provide more development ideas for the Rural-on-Demand-Oriented-bonded-GP medical graduates cultivation, and to consolidate the effectiveness of the government's policy implementation of Rural-on-Demand-Oriented-bonded-GPs and to provide a reference frame. | | First batch of Rural-on-Demand-Oriented-bonded-GP undergraduate students in Zhejiang | | 91 |
| 17 | 2019 | ^*^Investigation and analysis on occupational cognition of undergraduates majoring in general medicine [practice][49] | | Questionnaire designed by the authors after literature review and consulting experts: (1) demographics and (2) understanding of GP as a career, career prospects and career intentions | ^*^To investigate the occupational cognition of undergraduates majoring in general practice and to provide theoretical basis and data reference for the comprehensive construction of general medicine [practice] education system. | | General practice students studying clinical courses in a medical university | | 34 |
| 18 | 2020 | ^*^Investigation on influence of general family medicine education on employment direction of students in a medical college[30] | | ^*^A total of 400 freshmen in the clinical class were selected as a control group and 400 sophomores in the clinical class as the experimental group. A self⁃designed questionnaire was used to investigate the students’ awareness of general family medicine and general practitioners, and career orientation. The results were statistically analyzed and com⁃ pared between the two groups. | ^*^To investigate the change of employment thoughts among clinical students after general family medicine courses were offered in clinical classes. To judge the influence of general family education on students' career direction. | | 800 clinical medicine students of classes 2014 and 2015 in Shangqiu Medical College | | 800 |
| 19 | 2016 | Investigation on the recognition and employment intention of the course “General Medicine [practice] Introduction” in a Chinese medicine school[35] | | online survey | To understand the Chinese Medicine medical students’ perception on the “Introduction to General Practice” course and their employment intentions after graduation and the influencing factors. | | Students enrolled in “Introduction to General Practice” course, including undergraduate students (five years training), and graduates students (seven or eight years training). | | 168 |
| 20 | 2019 | ^*^Analysis on occupational identity and influencing factors on rural demand-oriented medical students[46] | | Stratified cluster sampling method for questionnaire administration and qualitative interview.  ^*^The survey included basic information, willingness to work at the grassroots level, reasons for applying for the profession and professional pride and other aspects of evaluation and recommendations. | ^*^To understand the recognition of rural demand‐oriented medical students in Yunnan province and put forward targeted suggestions and measures in combination with the actual situation to provide reference for the relevant departments to further standardize and improve the training of rural demand‐oriented medical students. | | Rural-on-Demand-Oriented-bonded-GP medical graduates | | 406 |
| 21 | 2019 | *Analysis on the present situation and influencing factors of grassroots employment intention in rural order-oriented medical students[42] | | ^*^The general information, intention of serving the grassroots employment intention and satisfaction degree of education service  quality in directional medical students selected by stratified cluster sampling method from a medical college were investigated using  questionnaire. | ^*^To investigate the employment intention rural order-oriented medical students at grassroot and analyse the influencing factors related to choosing general practitioners as a career for providing advice for training targeted medical students. | | All students studying general practice in a medical college: Year 1 = 60 Year 2 = 61 Year 3 = 50 Year 4 = 52 Year 5 = 55 | | 278 |
| 22 | 2019 | ^*^Investigation and analysis on general practice cognition and teaching guidance of medical students in colleges and universities[27] | | Survey designed by authors: (1) demographics, (2) understanding and learning channels of general practice, (3) evaluation of “introduction to general practice” course, (4) understanding of GP as a career and (5) interests and career planning in general practice | ^*^To fully understand the cognitive degree [level of understanding], curriculum evaluation and career choice of medical students in colleges and universities. To explore and construct a perfect [better] curriculum system of general medicine [practice] in colleges and universities, so as to cultivate [more] better quality general medicine [practice] talents for our country. | | Junior clinical trainee students in Year 3. | | 122 |
| 23 | 2016 | Investigation on medical students' perception and career intention of general practice in a medical university[36] | | Stratified random sampling for survey: (1) understanding of general practice and (2) career intentions | To investigate medical students’ understandings of general practice, career intentions, and influencing factors of choosing GP as a career, in order to provide suggestions to general practice education and development. | | Medical students (n=500) | | 356 clinical medicine major students, 122 general practice major students |
| 24 | 2019 | ^*^Barriers for general practitioner trainees becoming registered general practitioners: a survey[28] | | ^*^Individual interviews on the topic of "negative factors associated with choosing general practice as a career"  were conducted in trainees selected by dynamic sampling until data saturation was reached. | ^*^To investigate the barriers for GP trainees becoming registered GPs, and to make corresponding recommendations for government decision-making of improvement strategies. | | Participants were GP trainees who received general practice theoretical training in The Second Affiliated Hospital of Nanjing Medical University in October 2018 GP trainees “5+3” (*n=*12), “3+2” trainees (*n*=8), job-transfer trainees (*n*=5) | | 25 |
| 25 | 2016 | ^*^Investigation of the cognition of speciality and career intention of undergraduates in general practice medicine[51] | | ^*^The cognition of speciality and career intention of GP undergraduates from Bengbu Medical College were investigated using questionnaire survey. | ^*^To investigate the cognition of speciality and career intention of undergraduates in general practice. | | Bengbu Medical College GP major 252 undergraduate students | | 245 |
| 26 | 2018 | Analysis on the present situation and influencing factors of primary care employment intention in rural-oriented medical students[48] | | Stratified cluster sampling method for questionnaire: (1) demographics, (2) intention to work at primary care and (3) satisfaction of education and service quality. | To investigate the employment intention of rural order-oriented medical students at primary care and analyse the influencing factors related to choosing general practitioners as a career for providing advice for training targeted medical students. | | General practice-major students: Year 1 = 60 Year 2 = 61 Year 3 = 50 Year 4 = 52 | | 223 |
| 27 | 2018 | ^*^Study on the willingness and influencing factors of medical undergraduates to apply for general practitioners[24] | | Convenience sampling method, questionnaire designed by the authors | ^*^To understand medical undergraduates’ attitudes and the willingness to apply for general practitioners and to analyse the influencing factors so as to guide medical undergraduates to correctly understand general practice and provide evidence for the formulation of policies and measures in Guangzhou. | | 2012 to 2014 cohorts of clinical medical students (*n* = 420). | | 359  Year 3: 78 (21.7%), Year 4: 179 (49.9%), Year 5: 102 (28.4%) |
| 28 | 2018 | ^*^General medical students career confidence index (GMSCCI) in China[25] | | ^*^The online survey  questionnaires were given to general medical students from 359 general practitioners’ standardized training communities and *Doctor*  *Online Magazine* readers between 20 May to 29 July 2016. All data of basic information, employment intension, the evaluation for the status quo and next 3 years’ development of general practitioners (including 6 dimensions and 12 variables: training system, personal value, income level, career development, social respect, professional risk) were included in the questionnaire. | ^*^To explore general medical students career confidence index (GMSCCI), and comprehensively quantify confidence of general medical students on general practitioners. | | Ten types of general practice medical students/trainees: 5-year clinical medicine (including traditional Chinese medicine) medical students, 3-year medical specialist medical students, 5-year Rural-on-Demand-Oriented-bonded-GP medical students, 3-year Standardized GP Training Programme Trainees, 2-year Standardized assistant GP Training Programme trainees, postgraduates in clinical medicine (general practice), trainees in Job-Transfer-to-GP Training for primary health personnel, trainees in on-the-job training of general practitioners for primary health personnel and trainees in other learning stages, and others. | | 768 |
| 29 | 2018 | ^*^Causes and countermeasures of disconnection between training and practice of general practitioners in China[50] | | Semi-structured interviews; compared findings with studies in other countries | To investigate the reasons why students enrolled in the training between 2012 and 2016 were unwilling to practice in primary medical and health institutions. Based on the results, to compare domestic versus foreign training models and related existing literature, in order to conduct in-depth analysis of the disconnection between GP training and GP practices. It also aims to propose strategies in order to further improve the GP training and primary care system. | | 45 students who enrolled in the “5+2+1” General Practice Master student training programme between 2012 and 2016. | | 45 |
| 30 | 2019 | ^*^Investigation and analysis of the first batch of graduated general practice trainees from large general hospitals in Henan Province[31] | | ^*^Anonymous questionnaires and written interviews were used to understand the situation of the GP training. The questionnaire mainly included five parts of the basic situation of GPs, the situation of GP residency, the understanding of general practice, the feedback of GP training, and the basic situation of GP’s employment. | ^*^This article tries to  find out the existing problems and causes, in order to provide a reference for better GP training in the future and gradually improve  the GP education, and to cultivate high-level GPs for the country. | | ^*^A total of 200 GPs who graduated in June of 2018  from the First Affiliated Hospital of Zhengzhou University were selected by cluster sampling from May to June in 2018. | | 200 |
| 31 | 2019 | ^*^Survey on employment intention of medical students in China[29] | | ^*^A self-administered questionnaire survey on the employment intention was conducted among grade-5 medical students of 5-year program in 8 medical schools from January to June  2017. 395 participants (161 males and 234 females) were selected by stratified random cluster sampling  Method. | ^*^To survey the employment intention of medical students graduated from five-year medicine program in China | | Year-5 medical students of 5-year program in 8 medical schools. | | 395 |
| 32 | 2020 | *Status of general practice residency training and career choice of trainees in Henan Province | | Self-administered questionnaire | ^*^To survey the status general practice residency training and career choice of trainees in Henan province. | | General practice residency training trainees (class of year 2014-2017) | | 332 |
| 33 | 2020 | *A survey on the status of the perception of General Practitioner occupation and employment intention of "5+3+ general practice resident trainees | | Survey and interview | *To understand the current situation of occupational perception and employment intention of “5+3” general practice resident trainees in a residential training base in Henan province by questionnaire, so as to analyse the problems existed in the process. Through the method of qualitative interview to understand the relevant factors affecting the occupational perception of general practice resident trainees, and put forward suggestions and improvement measures to provide reference basis for improving the training process of general practice residents in Henan province. | | “5+3” general practice resident trainees (class of year 2017-2019) | | 146 |
| 34 | 2021 | Investigation on General Practice Sequence Education Model for Traditional Chinese Medicine Undergraduate Clinical Students | | Survey | To understand the overall evaluation, cognition, and occupational influence of general practice courses by students majoring in TCM clinical medicine after implementing the sequential teaching model of general practice, and to explore the construction of a general practice teaching model with TCM characteristics. | | Undergraduate students of Traditional Chinese Medicine and Integrative Western and Chinese Medicine | | 117 |
| 35 | 2020 | *Investigation and Study on the Orientation and Service Willingness of TCM Students | | Survey and interviews | *To explore factors influencing the orientation of TCM students, and cultivate TCM talents who can serve primary health care institutions gladly and continuously. | | TCM Orientation Students | | 107 |
| 36 | 2020 | Status and Influencing Factors of General Practice Resident Doctors after Graduating from Standardized Training | | Qualitative interview and survey | To analyse the employment of trainees (resident trainees) post standardized training of general medicine residents in the Shaw Hospital affiliated to Zhejiang University School of Medicine and the influencing factors of their careers in general medicine, to provide a basis for further improvement of the training program of general practitioners. | | General practice resident trainees | | 63 |
| 37 | 2020 | Training and development issues of primary care general practitioners: City X in Henan as an example | | Qualitative interview and survey | To investigate the current training and development status of general practitioners in City X. | | GP standardized training trainers, trainees, non-GP doctors, community residents | | Trainers 2; GP program trainees 134, no-GP doctors 20, community residents 277 |
| 38 | 2020 | *Qualitative study on the factors influencing the practice intention of the trainees in standardized training of general practitioners in Shandong Province | | Qualitative study | *To investigate the general practitioners undergoing standardized training in Shandong province, to understand their willingness and considerations to continue engage in general practice, to analyse the existing problems, and to explore solutions to the existing problems so as to provide a basis for retaining general practitioners and stabilizing the team of general practitioners | | GP standardized training trainees | | 21 |
| 39 | 2020 | Influencing factors on intention to become GPs of clinical undergraduate students at Hebei Medical University and analysis of strategies | | Survey | To understand the current status of cognition, the degree of recognition of career development prospects, and the willingness to practice among the clinical medicine undergraduates of Hebei Medical University on the profession of general practitioners and the associated reasons and factors | | clinical medicine undergraduates | | 1064 |
| 40 | 2021 | Clinical Undergraduate Students' Career Intention to Work at Primary Care and Influencing Factors | | Survey | To analyse the understanding of general practice among clinical medicine undergraduates, their willingness to engage in community general practice work and the influencing factors, and to put forward constructive suggestions for the current situation of general practitioners | | clinical medicine undergraduates | | 1064 |
| English articles | | | | | | | | |  |
| 41 | 2016 | Attitudes towards primary care career in community health centres among medical students in China[32] | | Cross sectional survey | ^*^The objective of this study is to examine the factors that affect medical students’ career choice as general medicine [practice] and contribute to the current knowledge of the attitudes of medical students towards working in primary care settings. | | Medical students | | 2402 |
| 42 | 2018 | Health‐personnel recruitment and retention target policy for health care providers in the rural communities: A retrospective investigation at Pudong New Area of Shanghai in China[43] | | Policy review/analysis | ^*^The aim of the current investigation was to probe into the related incentive measures, exploring the initial effect, so as to use the current retrospective study as a reference for other rural areas where there exist a shortage of health personnel and insufficient medical skill. | | GPs, physicians, nurses, the health technicians | | 964 GPs |
|  |  |  | |  |  | |  | |  |
| 43 | 2021 | Who is willing to participate in and provide Family Doctor Contract Service? | | Anonymous self-administered survey was conducted among the medical staff from tertiary hospitals | This study aims to explore the factors affecting the willingness of family doctor contracting from the tertiary hospital medical staff’s perspective. | | Medical staff in tertiary hospitals in Hangzhou, Zhejiang | | 346 |
| 44 | 2021 | Early outcomes of a rural-oriented physician education programme against rural physician shortages in Guangxi province: a prospective cohort study | | A prospective cohort study consisting of a baseline investigation and follow- up research | This study aimed to investigate early outcomes of one of the first medical undergraduate education programmes with a goal of mitigating severe rural physician shortages in China, which was developed by Guangxi Medical University (GXMU) and was called the Rural- oriented Free Tuition Medical Education (RTME)- GXMU programme. | | Rural-Oriented Free Tuition Medical Education (RTME) and non-RTME graduates at the Guangxi Medical University | | 380 RTME and 383 non-RTME graduates |
| 45 | 2021 | General practitioner trainees’ career perspectives after COVID-19: a qualitative study in China | | Semi-structured interviews | To explore Chinese GP trainees’ career perspectives after COVID-19 | | GP trainees | | 12 |
